# Supplementary material for: Multi‐dataset identification of innovative feature genes and molecular mechanisms in keratoconus
Source: J Cell Mol Med. 2024 Sep 19;28(18):e70079. doi: 10.1111/jcmm.70079 (PMC11412914; doi:10.1111/jcmm.70079)
Supplement: Supplementary file 2 — Material S2. [file JCMM-28-e70079-s002.zip › 7.GSEA analysis/4.MYRF/KEGG_EPITHELIAL_CELL_SIGNALING_IN_HELICOBACTER_PYLORI_INFECTION.html]

Details for gene set KEGG\_EPITHELIAL\_CELL\_SIGNALING\_IN\_HELICOBACTER\_PYLORI\_INFECTION[GSEA]

|  || Dataset | MYRF.group.cls#h\_versus\_l |
| Phenotype | group.cls#h\_versus\_l |
| Upregulated in class | l |
| GeneSet | KEGG\_EPITHELIAL\_CELL\_SIGNALING\_IN\_HELICOBACTER\_PYLORI\_INFECTION |
| Enrichment Score (ES) | -0.55072427 |
| Normalized Enrichment Score (NES) | -1.9224135 |
| Nominal p-value | 0.006185567 |
| FDR q-value | 0.04783109 |
| FWER p-Value | 0.033 |
Table: GSEA Results Summary

  

Fig 1: Enrichment plot: KEGG\_EPITHELIAL\_CELL\_SIGNALING\_IN\_HELICOBACTER\_PYLORI\_INFECTION      
 Profile of the Running ES Score & Positions of GeneSet Members on the Rank Ordered List

  

| SYMBOL | TITLE | RANK IN GENE LIST | RANK METRIC SCORE | RUNNING ES | CORE ENRICHMENT || 1 | ATP6V0A4 | na | 3203 | 0.285 | -0.0653 | No |
| 2 | CXCR2 | na | 3923 | 0.257 | -0.0617 | No |
| 3 | ATP6V0D2 | na | 5393 | 0.211 | -0.0841 | No |
| 4 | MAPK9 | na | 8409 | 0.129 | -0.1589 | No |
| 5 | MAPK12 | na | 8572 | 0.125 | -0.1517 | No |
| 6 | CASP3 | na | 8824 | 0.120 | -0.1476 | No |
| 7 | ATP6V1G3 | na | 9641 | 0.103 | -0.1613 | No |
| 8 | MAPK11 | na | 10606 | 0.085 | -0.1811 | No |
| 9 | MAPK13 | na | 10951 | 0.079 | -0.1836 | No |
| 10 | PTPN11 | na | 11569 | 0.069 | -0.1948 | No |
| 11 | MAPK14 | na | 11965 | 0.064 | -0.2001 | No |
| 12 | MAPK10 | na | 12440 | 0.057 | -0.2084 | No |
| 13 | PLCG1 | na | 12816 | 0.052 | -0.2142 | No |
| 14 | TJP1 | na | 12904 | 0.051 | -0.2119 | No |
| 15 | PLCG2 | na | 12987 | 0.050 | -0.2095 | No |
| 16 | IKBKB | na | 12994 | 0.050 | -0.2049 | No |
| 17 | MAP2K4 | na | 13264 | 0.047 | -0.2082 | No |
| 18 | ATP6V0D1 | na | 13308 | 0.046 | -0.2051 | No |
| 19 | CSK | na | 13727 | 0.041 | -0.2132 | No |
| 20 | ATP6V0E2 | na | 13911 | 0.039 | -0.2148 | No |
| 21 | CHUK | na | 14348 | 0.034 | -0.2242 | No |
| 22 | ATP6V1D | na | 14591 | 0.032 | -0.2282 | No |
| 23 | ATP6V1B1 | na | 14955 | 0.028 | -0.2359 | No |
| 24 | ATP6AP1 | na | 15209 | 0.026 | -0.2408 | No |
| 25 | ATP6V0A1 | na | 15296 | 0.025 | -0.2409 | No |
| 26 | ATP6V1B2 | na | 16165 | 0.016 | -0.2644 | No |
| 27 | ATP6V0B | na | 16214 | 0.016 | -0.2643 | No |
| 28 | JUN | na | 16671 | 0.011 | -0.2764 | No |
| 29 | EGFR | na | 17031 | 0.008 | -0.2860 | No |
| 30 | ATP6V0E1 | na | 17244 | 0.006 | -0.2915 | No |
| 31 | ATP6V1C1 | na | 17389 | 0.005 | -0.2952 | No |
| 32 | ATP6V1F | na | 17480 | 0.004 | -0.2973 | No |
| 33 | F11R | na | 18606 | -0.006 | -0.3292 | No |
| 34 | MAPK8 | na | 18612 | -0.006 | -0.3287 | No |
| 35 | ATP6V0C | na | 19202 | -0.013 | -0.3445 | No |
| 36 | ATP6V1E1 | na | 19370 | -0.015 | -0.3480 | No |
| 37 | GIT1 | na | 20104 | -0.023 | -0.3669 | No |
| 38 | ATP6V1A | na | 20121 | -0.023 | -0.3651 | No |
| 39 | ADAM10 | na | 20986 | -0.033 | -0.3869 | No |
| 40 | IKBKG | na | 21687 | -0.042 | -0.4031 | No |
| 41 | RAC1 | na | 21936 | -0.045 | -0.4060 | No |
| 42 | ATP6V1G1 | na | 22235 | -0.049 | -0.4100 | No |
| 43 | NOD1 | na | 23048 | -0.060 | -0.4277 | No |
| 44 | ATP6V1H | na | 23208 | -0.063 | -0.4263 | No |
| 45 | CDC42 | na | 23297 | -0.064 | -0.4228 | No |
| 46 | ATP6V1C2 | na | 25476 | -0.102 | -0.4759 | No |
| 47 | ADAM17 | na | 25659 | -0.106 | -0.4712 | No |
| 48 | PAK1 | na | 26325 | -0.120 | -0.4790 | No |
| 49 | ATP6V1E2 | na | 27833 | -0.156 | -0.5077 | No |
| 50 | NFKB1 | na | 28241 | -0.168 | -0.5036 | No |
| 51 | MET | na | 28755 | -0.183 | -0.5010 | No |
| 52 | LYN | na | 28768 | -0.184 | -0.4839 | No |
| 53 | CCL5 | na | 31086 | -0.268 | -0.5253 | Yes |
| 54 | RELA | na | 31135 | -0.271 | -0.5010 | Yes |
| 55 | SRC | na | 31183 | -0.273 | -0.4765 | Yes |
| 56 | NFKBIA | na | 31525 | -0.292 | -0.4587 | Yes |
| 57 | IGSF5 | na | 31655 | -0.298 | -0.4341 | Yes |
| 58 | HBEGF | na | 31666 | -0.299 | -0.4060 | Yes |
| 59 | MAP3K14 | na | 32615 | -0.359 | -0.3994 | Yes |
| 60 | ATP6V1G2 | na | 33275 | -0.416 | -0.3789 | Yes |
| 61 | PTPRZ1 | na | 33923 | -0.508 | -0.3494 | Yes |
| 62 | JAM2 | na | 34223 | -0.578 | -0.3033 | Yes |
| 63 | CXCL1 | na | 34529 | -0.693 | -0.2464 | Yes |
| 64 | CXCR1 | na | 34635 | -0.792 | -0.1744 | Yes |
| 65 | CXCL8 | na | 34657 | -0.838 | -0.0956 | Yes |
| 66 | JAM3 | na | 34714 | -1.030 | 0.0004 | Yes |
Table: GSEA details [plain text format]

  

Fig 2: KEGG\_EPITHELIAL\_CELL\_SIGNALING\_IN\_HELICOBACTER\_PYLORI\_INFECTION      
 Blue-Pink O' Gram in the Space of the Analyzed GeneSet

  

Fig 3: KEGG\_EPITHELIAL\_CELL\_SIGNALING\_IN\_HELICOBACTER\_PYLORI\_INFECTION: Random ES distribution      
 Gene set null distribution of ES for **KEGG\_EPITHELIAL\_CELL\_SIGNALING\_IN\_HELICOBACTER\_PYLORI\_INFECTION**
